# Dysregulated proteome and N-glycoproteome in ALG1-deficient fibroblasts

**DOI:** 10.1002/pmic.202400012

**Published:** 2024-03-12

**Authors:** Rohit Budhraja, Neha Joshi, Silvia Radenkovic, Tamas Kozicz, Eva Morava, Akhilesh Pandey

**Affiliations:** 1 Department of Laboratory Medicine and Pathology, Mayo Clinic, Rochester, Minnesota, USA; 2 Manipal Academy of Higher Education, Manipal, Karnataka, India; 3 Department of Clinical Genomics, Mayo Clinic, Rochester, Minnesota, USA; 4 Center for Individualized Medicine, Mayo Clinic, Rochester, Minnesota, USA

**Keywords:** ALG1-CDG, biomarker, congenital disorders of glycosylation, glycoproteomics, LC-MS/MS, proteomics

## Abstract

Asparagine-linked glycosylation 1 protein is a *β*-1,4-mannosyltransferase, is encoded by the *ALG1* gene, which catalyzes the first step of mannosylation in N-glycosylation. Pathogenic variants in *ALG1* cause a rare autosomal recessive disorder termed as ALG1-CDG. We performed a quantitative proteomics and N-glycoproteomics study in fibroblasts derived from patients with one homozygous and two compound heterozygous pathogenic variants in *ALG1*. Several proteins that exhibited significant upregulation included insulin-like growth factor II and pleckstrin, whereas hyaluronan and proteoglycan link protein 1 was downregulated. These proteins are crucial for cell growth, survival and differentiation. Additionally, we observed a decrease in the expression of mitochondrial proteins and an increase in autophagy-related proteins, suggesting mitochondrial and cellular stress. N-glycoproteomics revealed the reduction in high-mannose and complex/hybrid glycopeptides derived from numerous proteins in patients explaining that defect in *ALG1* has broad effects on glycosylation. Further, we detected an increase in several short oligosaccharides, including chitobiose (HexNAc_2_) trisaccharides (Hex-HexNAc_2_) and novel tetrasaccharides (NeuAc-Hex-HexNAc_2_) derived from essential proteins including LAMP1, CD44 and integrin. These changes in glycosylation were observed in all patients irrespective of their gene variants. Overall, our findings not only provide novel molecular insights into understanding ALG1-CDG but also offer short oligosaccharide-bearing peptides as potential biomarkers.

## 1 Introduction

Congenital disorders of glycosylation (CDG) are a group of inherited genetic disorders comprising over 150 genes. Pathogenic variants in these genes result in defective protein and lipid glycosylation. Asparagine-linked glycosylation 1, encoded by *ALG1* gene (MIM# 605907), is a chitobiosyldiphosphodolichol *β*-1,4-mannosyltransferase which catalyzes the transfer of the first mannose of the nine-mannose glycan structure onto the growing dolichol lipid-linked oligosaccharides required for N-linked glycosylation [1, 2]. In cases where the ALG1 enzyme is not functional, the full Glc_3_Man_9_GlcNAc_2_ oligosaccharide structure cannot be synthesized on the dolichol lipid precursor [3–5]. This leads to the accumulation of incompletely assembled glycan structures and results in the hypoglycosylation of glycoproteins [1, 6]. This deficiency in N-glycans is responsible for the development of ALG1-CDG [3–5]. ALG1-CDG is an autosomal recessive disease that presents with mild to severe neurological symptoms such as psychomotor developmental delay, intellectual disability, hypotonia, and seizures along with multi-organ system involvement mostly dysmorphism, respiratory and gastrointestinal difficulties [1, 7, 8].

The suspicion of ALG1-CDG is routinely made based on the clinical presentation and detailed patient history. Although the serum transferrin analysis is deployed to screen for the disease, the definitive diagnosis of ALG1-CDG is done through molecular genetic testing. Most patients except for one in the literature showed abnormal transferrin glycosylation [1, 7–9]. With an increase in whole exome and whole genome sequencing to investigate complex neurologic disorders in children, more and more variants in the *ALG1* gene are being reported necessitating additional functional confirmation of the genetic variants. For facilitating a diagnosis of ALG1-CDG, along with carbohydrate deficient transferrin (CDT) assay and genetic testing, several studies pertaining to biomarkers in ALG1-CDG have been reported. A novel tetrasaccharide glycan unit comprised of Neu_5_Ac-Gal-GlcNAc_2_ that is released upon PNGaseF treatment was proposed as a potential marker based on identification from serum, plasma and patient-derived fibroblasts of ALG1-CDG and PMM2-CDG [10]. This novel tetrasaccharide was also detected on serum transferrin along with other glycoproteins that accumulate in patients with ALG1-CDG [10, 11]. However, this tetrasaccharide is not exclusive to ALG1-CDG and can also be found in PMM2-CDG patients. Further, there is a lack of information regarding the proteins to which this tetrasaccharide is linked.

While these studies have proposed the novel N-tetrasaccharide as a potential diagnostic marker for ALG1-CDG, the impact on global gene expression and cellular protein glycosylation in this CDG remains largely elusive. A deeper understanding and to find unique biomarkers for this disease can be provided by combining proteomics and glycoproteomics studies, which can reveal changes in cellular protein abundance, glycan composition, glycan heterogeneity, their attachment sites on proteins, and site occupancy on these proteins. The unique combination of different peptides and glycans obtained from glycoproteomics could provide potential biomarker(s) for ALG1-CDG.

In the current study, we performed high resolution mass spectrometry-based proteomic and glycoproteomic profiling of patient-derived fibroblasts in ALG1-CDG carrying different biallelic pathogenic variants in *ALG1*. The quantitative proteomics data revealed differential abundance of several proteins involved in various cellular processes, including autophagy and mitochondrial function. In-depth analysis of glycoproteins revealed an overall reduction of high-mannose and complex/hybrid type glycopeptides. In contrast, the glycopeptides bearing short oligosaccharides, such as a tetrasaccharide NeuAc-Hex-HexNAc_2_, a trisaccharide Hex-HexNAc_2_ and a chitobiose HexNAc_2_ exhibited an increase in ALG1-CDG. This study provides a comprehensive molecular insight into the proteome and glycoproteome alterations resulting from ALG1 deficiency. It unveils potential biomarkers for the clinical detection of ALG1-CDG and offers guidance for future research aimed at deciphering the biological implications and dysregulation of glycosylation in ALG1-CDG.

## 2 Materials And Methods

### 2.1 Patient information and clinical data

Fibroblasts from three patients were included after informed consent under institutional approval (IRB: 19-005187). Of these, two were male pediatric patients, while one was female, with ages ranging from 11 to 17 years. The patients carried different biallelic pathogenic variants. One patient was homozygous (p.S258L/p.S258L) while the other two were compound heterozygous (p.A360V,—and p.Q50R/p.S258L) in the *ALG1* gene (Table 1). We also evaluated the clinical phenotypes and laboratory abnormalities of these three patients from 208 enrolled CDG patients followed by the Frontiers of Congenital Disorders of Glycosylation Consortium (FCDGC) natural history study (IRB: 19-005187; NCT04199000). Prospective data, as well as retrospective data, were available for all patients as part of standard care at the study site. This study was performed on only three sets of patient-derived fibroblasts owing to the rare nature of this inherited genetic disorder.

All patients presented with classic ALG1-CDG-related clinical features, including developmental delay, intellectual disability, hypotonia and seizures (Table 1). Dysmorphic features and skeletal abnormalities were variable. Hematologic abnormalities included decreased antithrombin activity and decreased factor XI activity. Patients had elevated transaminase levels in blood but showed no synthetic liver dysfunction. Patient 1 had significant proteinuria. No unique clinical features were noted in the three patients. None of the patients had hepatomegaly, or cardiac involvement. Carbohydrate deficient transferrin was increased in all cases. All detected pathogenic variants in the cohort have been published previously [1].

### 2.2 Cell culture

Three age-matched control fibroblasts (GM5400, GM5381 and GM5757) were obtained from Coriell Institute. All fibroblast cultures were maintained at sub confluent densities in RPMI-1640 Medium (Gibco) containing 0.5 mM glucose and 10% Fetal Bovine Serum (FBS; Gibco) supplemented with antibiotics and non-essential amino acids maintained in an incubator at 37°C with 5% CO_2_. Cells were harvested by scraping in phosphate-buffered saline (PBS, pH 7.4) and centrifuged at 2000 rpm at 4°C for 10 min.

### 2.3 Cell lysis and protein digestion

Cell pellet was solubilized in 8 M urea (in 100 mM TEAB buffer) supplemented with 1% protease inhibitor cocktail (Thermo Scientific). Cells were then sonicated with a tip sonicator at 30% amplitude for 3 cycles of 10 s each. Protein amount was estimated by BCA assay as per the manufacturer’s instructions (Thermo Scientific). Equal amounts of protein from both patient and control fibroblasts were first reduced with 10 mM dithiothreitol (Sigma-Aldrich, USA) at 37°C with mild shaking on thermomixer followed by alkylated with 40 mM iodoacetamide (Sigma-Aldrich, USA) at room temperature in dark. The proteins were then digested with 1:20 w/w (trypsin:protein) ratio (Worthington, USA) at 37°C overnight with mild shaking on thermomixer. Resulting peptides were cleaned up using C_18_ tips (Glygen, USA) and labeled with tandem mass tags (TMT) pro (Thermo Fisher Scientific, USA) as per the manufacturer’s protocol. The scheme of TMT labeling is provided in Table S1.

### 2.4 Peptide fractionation

First, the TMT labeling efficiency was checked in labeled peptides and samples were pooled after normalization. Pooled peptides were subsequently split into two aliquots. One aliquot containing about 20% of total peptides was resuspended in solvent A (5 mM ammonium formate, pH 9) and fractionated by basic pH reversed phase liquid chromatography (bRPLC) on a C_18_ column (5 μm, 4.6 × 100 mm column, Waters) using a linear gradient of solvent B (5 mM ammonium formate, pH 9, in 90% acetonitrile) for 120 min on the Ultimate 3000 UHPLC system. Ninety-six fractions were collected and subsequently concatenated into 12 fractions for the proteomics study. These concatenated 12 fractions were lyophilized and resuspended in 0.1% formic acid for liquid chromatography-tandem mass spectrometry (LC-MS/MS) analysis.

### 2.5 Glycopeptide enrichment

The other aliquot (remaining 80% peptides) was resuspended in 0.1% formic acid and injected into Superdex peptide 10/300 column (GE Healthcare) as described previously [12]. The peptides were separated using an isocratic flow of 0.1% formic acid for 130 min and 24 early fractions were collected starting at 10 min after injection which were concatenated into 12 fractions for glycoproteomics study. The fractions were lyophilized and resuspended in 0.1% formic acid for LC-MS/MS analysis.

### 2.6 Liquid chromatography tandem mass spectrometry (LC-MS/MS)

LC-MS/MS analysis of fractionated samples from both proteomics and glycoproteomics was carried out as previously described with some modifications [13, 14]. Briefly, 12 concatenated fractions from bRPLC and 12 concatenated fractions from SEC were analyzed on an Orbitrap Eclipse mass spectrometer (Thermo Fisher Scientific). Peptides were separated by liquid chromatography on an EASY-Spray column (75 μm × 50 cm, PepMap RSCL C_18_, Thermo Fisher Scientific). Peptides were first trapped on a trap column (100 mm × 2 cm, Acclaim PepMap100 Nano-Trap, Thermo Fisher Scientific) at a flow rate of 20 μL/min. LC separation was performed at a flow rate of 300 nL/min for 150 min using a linear gradient of 0.1% formic acid in water (solvent A) and 0.1% formic acid in acetonitrile (solvent B). All experiments were done in DDA mode at an isolation window of 0.7 m/z. Precursor ions were acquired at a resolution of 120,000 (at m/z 200) and fragment ions at a resolution of 30,000 (at m/z 200). Data acquisition was performed with option of “lock mass” (m/z 445.12002) for all data. Precursor fragmentation was carried out using normalized higher-energy collisional dissociation (HCD) method of 34 for proteomics and normalized stepped HCD at 15%, 25%, and 40% for glycoproteomics. The stepped HCD provides information about both glycan and peptide moieties. The scans were acquired in top-speed method with 3 s cycle time between MS and MS/MS.

### 2.7 Data analysis

Data analysis was performed as described previously [13]. The proteomics data were searched using Sequest search engine in Proteome Discoverer 2.5 against the human UniProt protein database (20 432 entries). The glycoproteomics data was searched using the publicly available software pGlyco3, which uses a built-in human N-glycan database containing 2922 entries [15]. We manually added HexNAc, HexNAc_2_, Hex-HexNAc_2_, Hex_2_-HexNAc_2_, HexNAc-Fuc, HexNAc_2_-Fuc, Hex-HexNAc_2_-Fuc, Hex_2_-HexNAc_2_-Fuc, and NeuAc-Hex-HexNAc_2_-Fuc N-glycan compositions to the pGlyco3 N-glycan database. A complete list of all N-glycans used to perform the analysis is provided as Table S1. We used the updated N-glycan database to search the N-glycoproteomics data for identifying N-glycans and human UniProt protein database (20,432 entries) for identifying peptide sequences. Two missed cleavages were allowed for both proteomics and glycoproteomics analysis. Error tolerance for precursor and fragment ions were set to 10 ppm and 0.02 Da, respectively, for proteomics and 10 and 20 ppm, respectively, for glycoproteomics. Cysteine carbamidomethylation was set as fixed modification, whereas oxidation of methionine and deamidation of asparagine and glutamine as variable modification. False discovery rate (FDR) was set to 1% at the peptide-spectrum matches (PSMs), peptide, protein, and glycopeptides levels. The FDR for all identified glycopeptides were controlled by pGlyco software, where the FDR threshold for all glycan species, peptide backbones and glycopeptides was set to 1%. For proteomics, quantitation of peptides across ALG1-CDG and control fibroblasts was done using TMT reporter ion intensities using “reporter ion quantifier” node. To quantify glycopeptides, reporter ion quantification was performed for glycoproteomics raw files in Proteome Discoverer and glycopeptide IDs obtained from pGlyco3 were matched with quantitation on a scan-to-scan basis (MS/MS). The proteomics data was searched against the annotated gene list for autophagy generated from Gene Ontology (GO) resources. For mitochondrial proteins, annotated gene list was generated from MitoCarta 3.0 [16].

### 2.8 Statistical analyses

Fold-changes of proteins and glycopeptides were calculated as average values from affected individuals over average values from control samples. *p*-values were obtained using unpaired 2-tailed Student’s *t* test. A *p*-value less than 0.05 was considered significant. All data are expressed as mean ± SD. Statistical analysis for both proteomics and glycoproteomics data was performed using the publicly available computational platforms Perseus [17], MetaboAnalyst 5.0 [18]. Perseus was used to draw heatmaps and MetaboAnalyst 5.0 was used to draw principal component analysis score plots. To generate PCA plot, the data were first log transformed and mean normalized in MetaboAnalyst. R studio was used to generate differential chord diagram using the *circlize* package [19]. All dot plots were made using GraphPad Prism version 9.5.

## 3 Results

**Figure 1:**
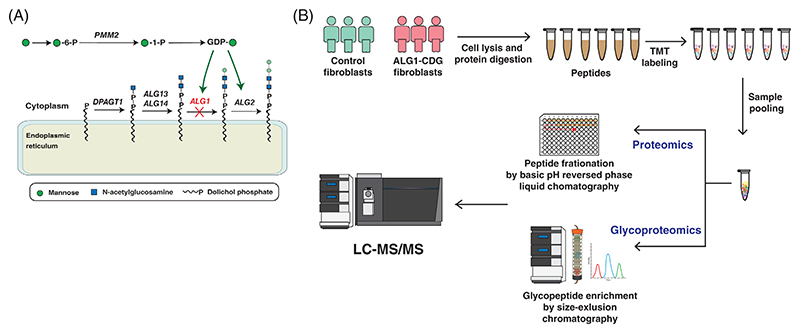
An overview of N-glycosylation biosynthetic pathway and schematic workflow for proteomic and glycoproteomic analyses of patient derived ALG1 fibroblasts. (A) Schematic representation showing the key steps involved in N-linked glycosylation pathway with the ALG1-dependent step highlighted with a red “X”. (B) The skin fibroblast cells from ALG1-CDG and control fibroblasts were lysed, proteins were extracted and digested with trypsin. Equal amounts of peptides were labeled with tandem mass tags (TMT) prior to pooling. Pooled TMT labeled samples were then split into two aliquots, where one aliquot was subjected to basic reversed-phase liquid chromatography (bRPLC) for proteomic analysis and the other part was used to enrich glycopeptides using size exclusion chromatography (SEC) for glycoproteomic analysis prior to LC-MS/MS.

Despite the crucial role of ALG1 in N-glycosylation, the impact of a defect in the *ALG1* on the cellular proteome and glycoproteome has not been investigated. In this study, we conducted a comprehensive analysis of ALG1-CDG patient-derived and control fibroblasts using a deep multiplexed approach encompassing both proteomics and glycoproteomics. We included three patients with three different genotypes (p.S258L/p.S258L and p.A360V, - and p.Q50R/p.S258L). Cells were lysed, proteins were extracted and subjected to in-solution proteolytic digestion. The resulting peptides from each sample were then labeled with tandem mass tags (TMT). The pooled labeled peptides were fractionated using bRPLC into 12 fractions for proteomics to reduce the complexity of cellular proteome [20, 21]. The other aliquot was used for the enrichment of glycopeptides using SEC because the glycopeptides are larger than non-glycosylated peptides [12, 13]. The enriched glycopeptides were concatenated into 12 fractions for glycoproteomics study. All these fractions were then analyzed by LC-MS/MS on an Orbitrap Eclipse mass spectrometer. A schematic of the optimized workflow for multiplexed proteomics and glycoproteomics is shown in Figure 1B.

### 3.1 Global proteomic alterations in ALG1 deficient fibroblasts

**Figure 2:**
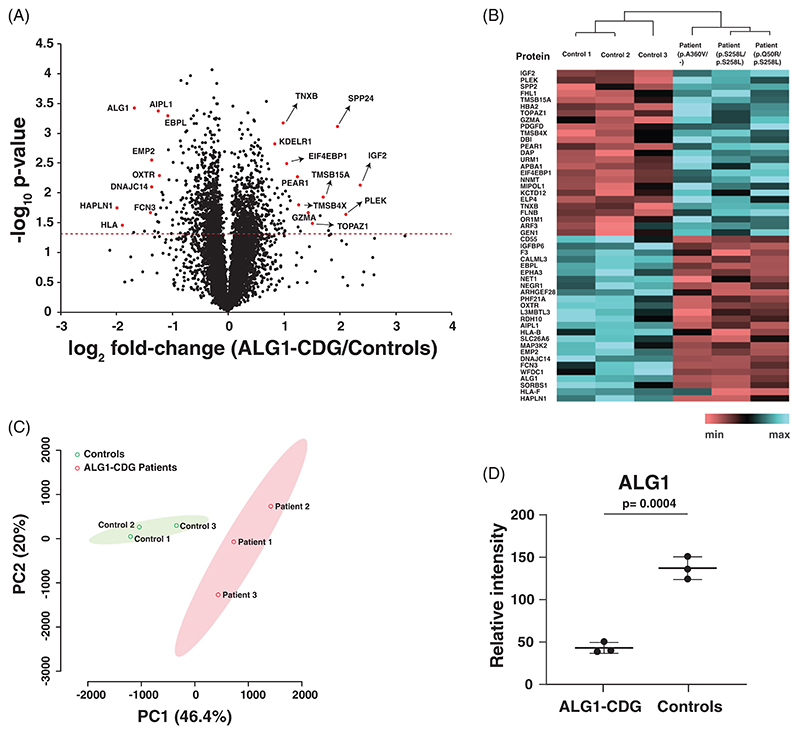
Quantitative proteomic changes in patient derived ALG1 fibroblasts. (A) Volcano plot depicting the differentially abundant proteins in ALG1-CDG patients. X-axis is log_2_ fold-change (ALG1-CDG/controls) and Y-axis is the negative logarithm of the p-value from a t test for significance as indicated. The horizontal dashed red line represents the cutoff for significance (<0.05). Some of the highly changing proteins are marked in red circles with the corresponding proteins indicated. (B) Heatmap of all significantly changing proteins showing the differential expression in ALG1-CDG patients. The genotypes for each patient are indicated. The pattern is color coded and gene symbols are given. (C) Principal component analysis (PCA) based on reporter ion intensities for all identified proteins of ALG1-CDG patients and controls. The percentage of total variance associated with each component is shown in brackets with the axis label. (D) Dot plot showing reporter ion intensities of ALG1 protein in ALG1-CDG patient fibroblasts. Y-axis is the reporter ion intensity of TMT channels.

We conducted a proteomics study on fibroblasts from individuals with ALG1-CDG due to the fact the glycosylation is essential for multiple cellular processes and deficiency in ALG1 might lead to alterations in the overall protein abundance within the cells. Multiplexed proteomics study of fibroblasts identified a total of 8368 proteins with 127,484 peptides. The volcano plot shows the distribution of protein expression, which was generated by using the average abundance of all patients and controls, demonstrating that ALG1-CDG patients exhibit the distinct expression of several proteins (Figure 2A). The top upregulated protein was insulin-like growth factor II (IGF2), which was increased by 5-fold in patient fibroblasts. Other proteins which were increased in patients by more than 2-fold included pleckstrin (PLEK), secreted phosphoprotein 24 (SPP24), four and a half LIM domains protein 1 (FHL1), thymosin beta-15A (TMSB15A), granzyme A (GZMA) and acyl-CoA-binding protein (DBI). Conversely, the top significantly downregulated protein was hyaluronan and proteoglycan link protein 1 (HAPLN1), which was decreased by 3.9-fold in patients. Among the other downregulated proteins, HLA class I histocompatibility antigen, alpha chain F (HLA-F), sorbin and SH3 domain-containing protein 1 (SORBS1), WAP four-disulfide core domain protein 1 (WFDC1), ficolin-3 (FCN3), epithelial membrane protein 2 (EMP2) and HLA class I histocompatibility antigen, B alpha chain (HLA-B) were downregulated by more than 2-fold. The expression patterns of significantly changing proteins across both patient and control groups are depicted in a heatmap showing their clustering within the groups (Figure 2B). Principal component analysis (PCA) also revealed separation between ALG1-CDG and control fibroblasts at the protein level (Figure 2C). Additionally, we observed a consistent global reduction of ALG1 protein levels by approximately 70% in all patient fibroblasts compared to controls (Figure 2D). A complete list of all significantly changing proteins interrogated with the fold-change and p-values can be found in Table S2.

### 3.2 Altered autophagy and mitochondria-related protein abundance in ALG1-CDG

**Figure 3:**
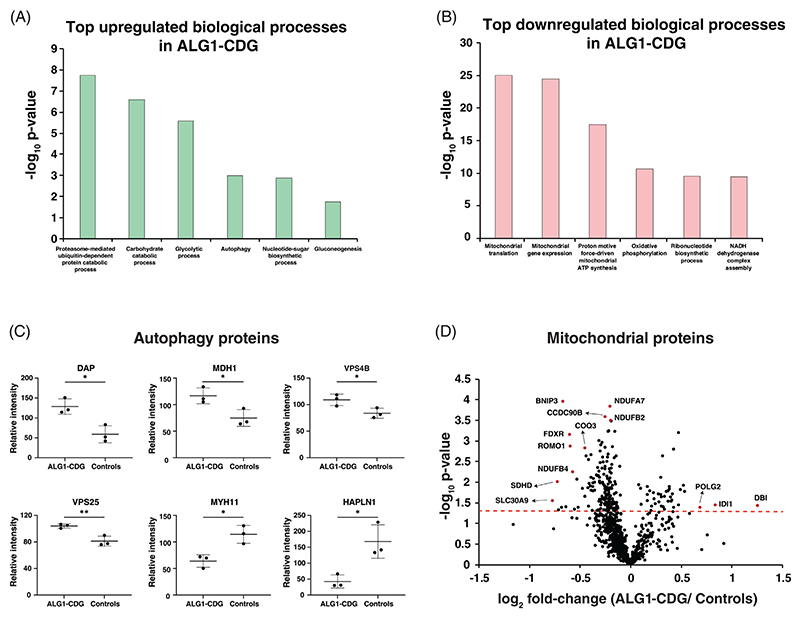
Gene ontology analysis of biological processes involving differentially expressed proteins. (A) Top upregulated enriched biological processes in ALG1-CDG patients. Y-axis is the negative logarithm of p-values for significance. (B) Top downregulated enriched biological processes in ALG1-CDG patients. Y-axis is the negative logarithm of p-values for significance. (C) Dot plots showing reporter ion intensities for top changing proteins related to autophagy. Y-axis is the reporter ion intensity of TMT channels. Each dot in the plots represent the individual control or patient sample. *p* < 0.05(*) and *p* < 0.01(**). (D) Volcano plot depicting the differentially abundant mitochondrial proteins in patient derived ALG1-deficient fibroblasts. X-axis is log_2_ fold-change (ALG1-CDG/controls) and Y-axis is the negative logarithm of p-value from a *t* test for significance as indicated. The horizontal dashed red line represents the cutoff for significance (<0.05). Some of the highly changing proteins are marked in red circles and protein names are provided.

We next performed Gene Ontology analysis of differentially expressed proteins to identify functionally relevant biological processes dysregulated in patient fibroblasts. Proteasome-mediated ubiquitin-dependent protein catabolic process, carbohydrate catabolic process, glycolytic process and autophagy were enriched among overexpressed proteins in ALG1-CDG (Figure 3A). In our previous studies, we also observed differential expression of autophagy-related proteins in other CDG, such as PMM2-CDG [22]. We observed the differential expression of multiple proteins which are annotated in autophagy process. Notably, the expression of death-associated protein 1 (DAP) was significantly increased showing a 2.1-fold upregulation in patients. The other autophagy pathways associated proteins which were increased in ALG1-CDG patients included malate dehydrogenase, cytoplasmic (MDH1), charged multivesicular body protein 2b (CHMP2B), platelet-activating factor acetylhydrolase IB subunit alpha2 (PAFAH1B2), vacuolar protein sorting-associated protein 4B (VPS4B), vacuolar protein-sorting-associated protein 25 (VPS25), ubiquilin-2, ubiquitin-like protein ATG12 (ATG12) and endophilin-B1 (SH3GLB1). These proteins are recognized for their role in promoting autophagy within cells. In contrast, proteins involved in cell growth and proliferation exhibited reduced expression in patient fibroblasts. These proteins included hyaluronan and proteoglycan link protein 1 (HAPLN1), myosin-11 (MYH11), transmembrane protein 59 (TMEM59) and dynein light chain 2 (DYNLL2). The expression of top changing autophagy-related proteins is represented in Figure 3C. The upregulation of autophagy-related proteins and the downregulation of proteins associated with cell proliferation in patient fibroblasts suggest that defects in N-glycosylation biosynthesis machinery could lead to cellular stress.

Downregulated proteins showed enrichment of several processes associated with mitochondrial function including mitochondrial translation, mitochondrial gene expression, proton motive force driven mitochondrial ATP synthesis, oxidative phosphorylation and NADH dehydrogenase complex assembly (Figure 3B). The downregulation of mitochondrial proteins was also observed in PMM2-CDG [22]. Among the 877 identified mitochondrial proteins, 40 proteins were significantly upregulated, and 173 proteins were significantly downregulated in ALG1-CDG patients with *p* < 0.05 (Figure 3D). Of particular significance, we observed a substantial decrease in multiple subunits of complex I (NADH: ubiquinone oxidoreductase) within patient fibroblasts. Additionally, several other mitochondrial proteins that were downregulated with more than 2-fold included zinc transporter 9 (SLC30A9), succinate dehydrogenase [ubiquinone] cytochrome b small subunit (SDHD), alpha-methylacyl-CoA racemase (AMACR), alanine aminotransferase 2 (GPT2) and NADPH: adrenodoxin oxidoreductase (FDXR). Downregulation of various mitochondrial proteins, including those associated with complex I and energy metabolism, underscores the stress experienced by mitochondria in these patients. Complete list of significantly changing proteins for autophagy and mitochondrial function interrogated with the fold-change can be found as Table S3.

### 3.3 ALG1 deficient fibroblasts exhibit distinct glycosylation remodeling

**Figure 4:**
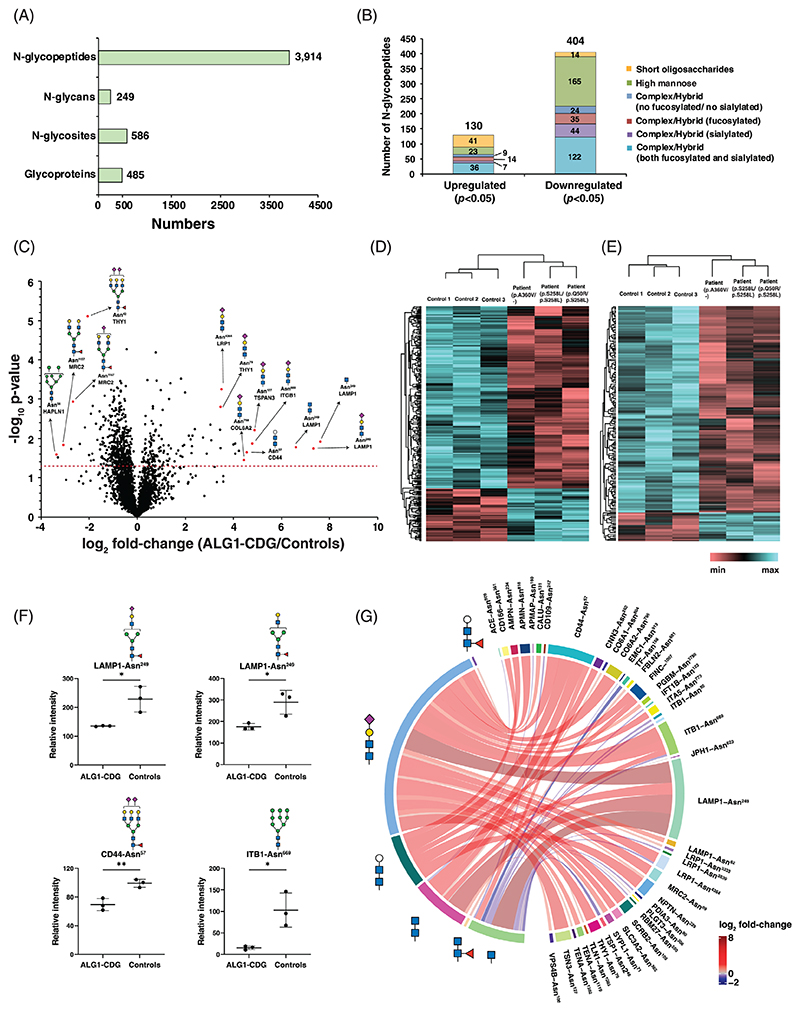
Site-specific glycosylation changes in patient derived ALG1-CDG fibroblasts. (A) A bar graph showing the global view of glycosylation in fibroblasts. (B) Glycopeptides that are significantly differentially expressed (*p* < 0.05) with different glycan classes are drawn in a stacked bar graph according to their relative expression in ALG1-CDG patients. (C) Volcano plot depicting the differentially expressed glycopeptides in ALG1-CDG patients. X-axis is log_2_ fold-change (ALG1-CDG/controls) and Y-axis is the negative logarithm of p-value from a t test for significance as indicated. The horizontal dashed red line represents the cutoff for significance (<0.05). Some of the changing glycopeptides are marked in red circles and glycoproteins’ names, glycosylation sites and glycan structures are drawn. Heatmap of significantly changing glycopeptides (*p*-value <0.05) with different (D) complex or hybrid glycan composition and (E) high-mannose glycan moieties. The genotypes for each patient are indicated. The pattern is color coded. (F) Dot plots showing reporter ion intensities of four downregulated regulated glycopeptides derived from LAMP1, CD44, and ITB1 in ALG1-CDG. Y-axis is the reporter ion intensity of TMT channels. Each dot in the plots represent the individual control or patient sample. (G) Differential chord diagram depicting the site-specific glycosylation changes for the proteins encompassing the oligosaccharides in ALG1-CDG as compared to control fibroblasts. Proteins with different glycosylation sites are indexed on the right of the diagram and connected via chords to respective identified glycan structures on the left. Asn^x^ represents the asparagine at amino acid site “x” in the corresponding protein sequence. The fold-change pattern is color coded. Putative structures are shown using Symbol Nomenclature for Glycans (SNFG). *p* < 0.05 (*), *p* < 0.01 (**).

In our global glycoproteomics study in skin fibroblasts, we identified and quantified 3,914 individual N-glycopeptides. These peptides encompassed 249 distinct N-glycan compositions and were located on 586 glycosylation sites associated with 485 glycoproteins (Figure 4A). In our recent investigations, we have consistently observed a substantial decrease in protein glycosylation levels in other CDG such as PMM2-CDG and PGM1-CDG [14, 22, 23]. We hypothesized that ALG1-CDG would likewise exhibit a decrease in N-glycosylation of multiple cellular proteins. In the current study, decreased abundance of N-glycopeptides was observed in all patient-derived ALG1 fibroblasts, affecting various glycosylation sites on multiple glycoproteins. Among the 3,914 identified glycopeptides, significant glycosylation change (*p* < 0.05) was observed in 534 unique N-glycopeptides derived from 163 distinct glycoproteins. Of these, 404 glycopeptides originating from 122 glycoproteins were reduced in patient derived ALG1 deficient fibroblasts compared to controls. These glycopeptides encompassed a wide range of glycan structures, including high mannose, complex/hybrid, sialylated, and fucosylated glycans. While glycopeptides with larger glycan structures displayed reduced expression, those with short oligosaccharides exhibited increased expression in ALG1-CDG patient fibroblasts. The expression of 41 glycopeptides with short oligosaccharides was increased in patients, whereas the expression of 14 glycopeptides was reduced (Figure 4B). The overall reduction in N-glycosylation is evident from the volcano plot shown in Figure 4C. We observed reduced expression of glycopeptides that encompass both high-mannose and complex/hybrid glycan structures in all three patients indicating that ALG1 deficiency results to a broader glycosylation abnormality in the cells (Figure 4D,E).

Among the most profoundly affected glycoproteins exhibiting a global reduction in glycosylation were prolow-density lipoprotein receptor-related protein 1 (LRP1; 33 glycopeptides with 15 glycan compositions from 13 glycosylation sites), integrin beta-1 (ITB1; 28 glycopeptides with 14 glycan compositions from 3 sites), aminopeptidase N (AMPN; 19 glycopeptides with 19 glycan compositions from 2 sites), thy-1 membrane glycoprotein (THY1; 16 glycopeptides with 12 glycan compositions from 2 sites), CD109 (CD109; 12 glycopeptides with 8 glycan compositions from 6 sites), tissue factor (TF; 10 glycopeptides with 10 glycan compositions from 1 site) and CD166 (CD166; 10 glycopeptides with 10 glycan compositions from 3 sites). Additionally, other dysregulated glycopeptides were derived from lysosome-associated membrane glycoprotein 1 (LAMP1), lysosome-associated membrane glycoprotein 2 (LAMP2), aspartyl/asparaginyl beta-hydroxylase (ASPH), beta-sarcoglycan (SGCB) and C-type mannose receptor 2 (MRC2). These changing glycopeptides exhibited varying glycan compositions ranging from paucimannose (Man_3_), high mannose (Man_4_ and higher) and complex/hybrid moieties. Relative expression of some of the significantly decreasing glycopeptides bearing mature form of glycan structure is shown in Figure 4F. A comprehensive list of significantly changing glycopeptides with glycosylation sites and glycan compositions interrogated with the fold-change and p-values can be found in Table S4.

### 3.4 Expression of short oligosaccharides derived glycopeptides in ALG1 deficient fibroblasts

Given that the ALG1 enzyme is essential for initiating the mannosylation in protein N-glycosylation biosynthetic pathway, we postulated that individuals with ALG1-CDG, may experience an accumulation of short oligosaccharides containing glycopeptides. Thus, we extended our glycoproteomics analysis to include a modified N-glycan database encompassing HexNAc and various smaller glycan structures as they are not detected normally. These smaller glycan structures included HexNAc, HexNAc_2_, Hex-HexNAc_2_, Hex_2_-HexNAc_2_, HexNAc-Fuc, HexNAc_2_-Fuc, Hex-HexNAc_2_-Fuc, Hex_2_-HexNAc_2_-Fuc, and NeuAc-Hex-HexNAc_2_-Fuc. Additionally, we incorporated a novel xeno -tetrasaccharide (NeuAc-Hex-HexNAc_2_) into the database, as previous studies had already identified this tetrasaccharide in the serum and fibroblasts of ALG1-CDG patients through released N-glycan analysis [10, 11]. In a recent investigation, this tetrasaccharide were also identified on several proteins in cerebrospinal fluid from patients with ALG1-CDG [24]. Our analysis revealed an upregulation in the expression of numerous glycopeptides containing these oligosaccharide structures in all patient-derived ALG1 deficient fibroblasts irrespective of the gene variants. A total of 41 unique glycopeptides derived from 26 glycoproteins containing such oligosaccharide compositions on different glycosylation sites were significantly increased (*p* < 0.05) in patients, whereas 14 glycopeptides derived from 11 glycoproteins were significantly downregulated. A differential chord diagram is drawn to quantitatively visualize glycosylation differences in patient as compared to control fibroblasts with different composition of oligosaccharide on distinct glycosylation sites of various cellular proteins (Figure 4G).

**Figure 5:**
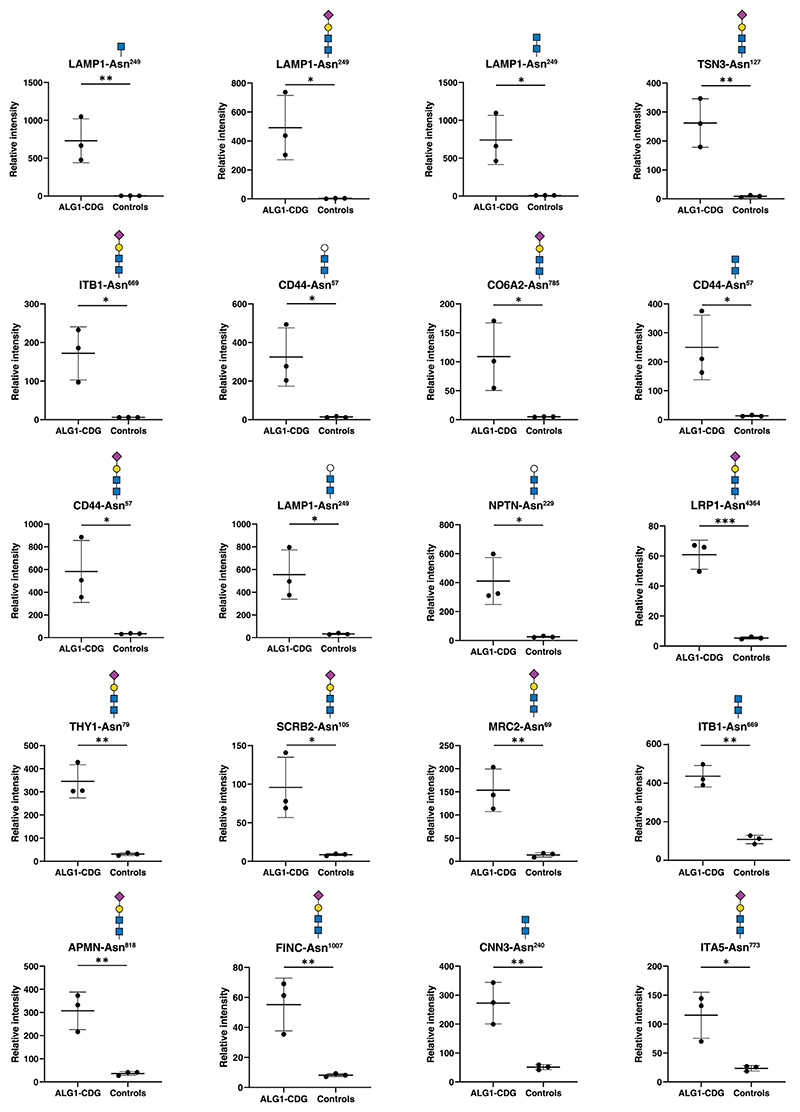
Site-specific alterations in abundance of various oligosaccharides bearing glycopeptides in ALG1-CDG. The dot plots depict the expression levels of glycopeptides associated with various oligosaccharides. Protein names, N-glycosylation sites and the oligosaccharide structures are indicated on the top of dot plots and X-axis depicts sample type while Y-axis is reporter ion intensity at the glycopeptide level. Asn^x^ represents the asparagine at amino acid site “x” in the corresponding protein sequence. Each dot in the plot corresponds to an individual sample from either the patient group or control. Putative structures are shown using Symbol Nomenclature for Glycans (SNFG). *p* < 0.05 (*), *p* < 0.01 (**), *p* < 0.001 (***).

Out of 41 glycopeptides that were overexpressed, 23 glycopeptides (from 19 different glycoproteins) contained glycans composed of NeuAc-Gal-GlcNAc_2_, consistent with earlier research findings. Noteworthy these glycoproteins were LAMP1, ITB1, LRP1, CD44, and tenascin (TENA), all possessing this glycan structure. Further, seven glycopeptides derived from seven different proteins featured a chitobiose (HexNAc_2_) composition, five glycopeptides derived from five different proteins contained a trisaccharide (Hex-HexNAc_2_) composition and 4 glycopeptides from 4 glycoproteins contained only a HexNAc. Given that *ALG1* gene deficiency affects early steps of N-glycosylation, it is highly likely that the structures HexNAc_2_ and HexNAc are actually GlcNAc_2_ and GlcNAc, respectively. Notably, among all upregulated glycopeptides, the top three were derived from LAMP1 on a single glycosylation site (Asn^249^), on which monosaccharide HexNAc was increased approximately 200-fold, xeno-tetrasaccharide (NeuAc-Gal-GlcNAc_2_) more than 150-fold and chitobiose (HexNAc_2_) about 100-fold in ALG1-CDG patients. The presence of a very low intensity signal at the m/z of respective TMT channels in controls may be the result of electronic noise or channel bleeding, which may account for the large fold-change for aberrant glycoforms instead of true infinity if absolutely no signals were detected in the control channels. Conversely, among glycopeptides with significantly reduced expression featuring these sugar structures, 12 glycopeptides carried a HexNAc residue across nine different glycoproteins, including three glycopeptides from the ITB1 glycoprotein. Unlike the increased expression of glycopeptides, the decrease of downregulated glycopeptides was relatively modest, typically ranging from 20% to 50%. Dot plots illustrating the differential expression of several top-changing glycopeptides with these specific sugar structures are presented in Figure 5. Representative MS/MS spectra for top changing glycopeptides from Figures 4F and 5 are shown in Figure S1.

## 4 Discussion

ALG1-CDG is a rare autosomal recessive disorder that results in multisystem dysfunction. There are 43 pathogenic variants reported in the *ALG1* gene (MIM# 605907) and the most common mutation is c.733C>T (p.Ser258Leu) [1, 25]. Of these known pathogenic variants, 29 are missense variants. In this study, our objective was to comprehensively analyze the global proteome and N-glycoproteome within fibroblasts derived from ALG1-deficient patients. This investigation employed a multiplexed mass spectrometry approach and encompassed a range of biallelic pathogenic variants in *ALG1*. Our aim was to gain insights into the pathophysiology of ALG1-CDG and to identify potential candidate biomarkers for diagnostic and therapeutic purposes. This is the first report describing alteration in proteome and glycoproteome in fibroblasts derived from ALG1-deficient patients.

Since glycosylation is essential for numerous functions including protein stability, cell proliferation, cell growth, energy metabolism and intracellular interactions, we conducted proteomics analysis along with glycoproteomics with the hypothesis that the ALG1 deficiency can also impact the abundance of several cellular proteins. The proteomics data reveal dysregulated abundance for several important cellular proteins. Among these proteins, insulin-like growth factor II (IGF2) emerged as the most prominently upregulated in patient fibroblasts. IGF2 is a well-known growth factor involved in cell proliferation, development and cell growth and its overexpression has been linked to multiple metabolic diseases including obesity and metabolic bone disease [26]. Another highly upregulated protein in ALG1-CDG patients was secreted phosphoprotein 24 kDa (SPP24), which is a bone matrix protein. SPP24 is a BMP-2 binding protein and this binding has been shown to inhibit the bone formation [27]. ALG1-CDG patients also showed reduced expression of several extracellular matrix proteins. The top downregulated protein, hyaluronan and proteoglycan link protein 1 (HAPLN1) has also been shown to be downregulated in PMM2-CDG [22] and in a congenital disorder of deglycosylation, namely NGLY1-CDDG [13] patient fibroblasts. HAPLN1 is a linker protein which stabilizes the proteoglycan-hyaluronan structures [28]. Notably, reduced levels of the ALG1 protein were consistently detected across all patient fibroblasts, irrespective of their specific gene variants.

The gene ontology analysis revealed dysregulation of several biological processes including mitochondrial functions and autophagy. Interestingly, in our previous report, we also observed dysregulated autophagy and mitochondria-associated proteins in PMM2-CDG fibroblasts [22]. We observed an elevation in the expression of autophagy related proteins in ALG1-CDG fibroblasts, supporting our hypothesis that the defect in N-glycan synthesis could lead to cellular stress. As observed in PMM2-CDG, death-associated protein 1 (DAP), endophilin B1 and several VSP and ATG proteins were also overexpressed in ALG1-CDG suggesting a shared mechanism of autophagy in different CDG. We also identified the reduced expression of multiple proteins known to participate in cell cycle regulation and cell growth. Our findings suggest compromised cellular bioenergetics in ALG1-CDG as we observed reduced expression of several mitochondrial proteins including several subunits of the respiratory complex I (NADH ubiquinone oxidoreductase) and succinate dehydrogenase [ubiquinone] cytochrome b small subunit in patients. Secondary mitochondrial dysfunction has also been reported in individuals with various other types of CDG [29–32].

Given that ALG1 plays an important role in the early stage of biosynthesis of N-glycosylation, we included truncated glycan species and previously reported glycan species in the N-glycan databases which are not present in pGlyco’s in-built N-glycan database. This modified N-glycan database required us to search the data against the entire human database and not against a sample specific glycoproteomics library. N-glycoproteomic profile in ALG1-CDG patient fibroblasts revealed significant glycosylation abnormalities. Specifically, we observed a marked reduction in mature high mannose, hybrid and complex type glycans, a pattern consistent with other type 1 CDG [14, 22, 23]. This global hypoglycosylation in patient fibroblasts is consistent with that fact that ALG1 catalyzes an early and crucial step in the N-glycosylation machinery in endoplasmic reticulum and the defect in *ALG1* disrupts the synthesis of various glycan classes. We detected diminished glycosylation on numerous extracellular matrix proteins and intracellular adhesion molecules such as HAPLN1, CADH6, LYOX, ITB1 and THY1 in which are known to be involved in development of central and peripheral nervous systems. Moreover, we identified various glycopeptides with distinct glycan classes on different glycosites of lysosome-associated membrane glycoprotein 1 (LAMP1) and lysosome-associated membrane glycoprotein 2 (LAMP2). The altered glycosylation for both proteins were also observed in PMM2-CDG fibroblasts [14]. These proteins are known regulators of autophagy, suggesting the dysregulation in autophagy process.

Previous studies have identified a novel xeno-tetrasaccharide (NeuAc-Gal-GlcNAc_2_) as a potential biomarker in ALG1-CDG patients. This discovery was made using a glycan release technique applied to serum and fibroblasts, coupled with N-glycan analysis. Additionally, it was identified on purified transferrin protein using MALDI-MS [10, 11]. The underlying hypothesis regarding the presence of this tetrasaccharide is that the flip of Dol-PP-GlcNAc_2_ from the cytoplasmic face into the ER luminal face. This structure is then transferred to the proteins and before transferring to Golgi where galactose residue is appended to GlcNAc_2_ followed by sialic acid forming a unique structure NeuAc-Gal-GlcNAc_2_ which can differentiate ALG1-CDG patients from control subjects. Nevertheless, the studies did not explore the occurrence of this oligosaccharide on other cellular proteins or the presence of other shorter oligosaccharides. However, in a recent study, this novel tetrasaccharide was detected on various proteins using glycoproteomics study in the cerebrospinal fluid samples from one ALG1-CDG patient [24]. We detected increased abundance of this novel tetrasaccharide and other shorter oligosaccharides including a trisaccharide (Hex-HexNAc_2_), a disaccharide (HexNAc_2_) and a monosaccharide (HexNAc) on several cellular proteins.

## 5 Conclusions

In summary, our findings provide evidence of significant changes in both protein levels and glycopeptide expression in patient derived ALG1-CDG fibroblasts. These observed differences in protein expression and the presence of hypoglycosylation in various glycopeptides hold promise for the identification of potential biomarkers for ALG1-CDG. Further, our research corroborates prior studies and confirms the existence of a unique xeno-tetrasaccharide in ALG1-CDG patients, shedding light on the specific cellular proteins harboring this oligosaccharide. We also report elevation of another shorter oligosaccharide-containing glycopeptides in these patients. The unique combination of these glycopeptide markers can be used in the future to confirm the diagnosis of suspected ALG1-CDG, especially in the absence of abnormal transferrin glycoforms in blood, or in individuals carrying variants of uncertain significance in ALG1. This comprehensive quantitative proteomics and cellular glycosylation profiling not only enhance our overall understanding of ALG1-CDG but also pave the way for novel avenues of research and targeted therapeutic interventions.

## Supplementary Material

**Supporting Information**

Additional supporting information may be found online https://doi.org/10.1002/pmic.202400012 in the Supporting Information section at the end of the article.

Table S1

Table S2

Table S3

Table S4

## Figures and Tables

**Table 1:** ALG1-CDG patients and their clinical features. List of included subjects with ALG1-CDG with demographic and genetic information. “+”: present. “−”: absent.

Patient	1	2	3
Sex	Male	Male	Female
Age (years)	11	17	12
Coding variant	c.773C>T/c.773C>T	c.1079C>T/ c.1187+1G>A	c.149A>G/c.773C>T
Protein change	p.S258L/p.S258L	p.A360V, -	p.Q50R/p.S258L
**Clinical features**			
Developmental delay	+	+	+
Intellectual disability	+	+	+
Hypotonia	+	+	+
Seizures	+	+	+
Visual involvement	−	−	+
Microcephaly	+	−	−
Abnormal brain imaging	Pontocerebellar hypoplasia	−	−
Facial dysmorphism	+	−	+
Hematological anomalies	+	+	+
Gastrointestinal problems	−	−	+
Skeletal anomalies	Short arms	−	−
Liver involvement	+	+	+
Edema	+	-	−
Cardiac involvement	−	−	−
Renal involvement	+	−	−
Low IgG levels	+	−	−

## Data Availability

The mass spectrometry proteomics data have been deposited with the ProteomeXchange Consortium via the PRIDE partner repository with the dataset identifier PXD044327. Reviewer account details: Username: reviewer_pxd044327@ebi.ac.uk; Password: lCqOFt75.

## Significance Statement

Asparagine-linked glycosylation 1 (ALG1) is a mannosyltransferase enzyme that catalyzes the first step of the mannosylation in dolichol-linked oligosaccharides biosynthesis. Defects in the *ALG1* gene affect both central and peripheral nervous systems. Despite the essential role of ALG1 in the glycosylation pathway, the exact consequences of ALG1 deficiency on global cellular proteome and their glycosylation have not yet been investigated. A comprehensive proteomic and glycoproteomics studies can help understand the biology and molecular mechanisms of ALG1-CDG. Additionally, besides proteins, alteration in the abundance of unique glycopeptides can serve as potential biomarker/s for ALG1-CDG. In this study, the combined proteomics and N-glycoproteomics revealed the altered protein levels in addition to a reduction of mature forms of glycopeptides in ALG1-CDG. These alterations are highly significant as they add a new link between biosynthesis of glycoproteins and cellular functions. Short oligosaccharide bearing glycopeptides from a variety of cellular proteins identified in this study could be used to differentiate different CDG types.

## Abbreviations

ALG1: asparagine-linked glycosylation 1
Asn^x^: asparagine at amino acid site x in a polypeptide sequence
bRPLC: basic pH reversed phase liquid chromatography
CDDG: congenital disorder(s) of deglycosylation
CDG: congenital disorder(s) of glycosylation
CDT: carbohydrate-deficient transferrin
Fuc: fucose
Gal: Galactose
Hex: hexose
HexNAc: N-acetylhexosamine
Man: mannose
NeuAc: N-acetylneuraminic acid
SEC: size exclusion chromatography
SNFG: Symbol Nomenclature for Glycans
TMT: tandem mass tag.
